# Automated Phasor Segmentation of Fluorescence Lifetime Imaging Data for Discriminating Pigments and Binders Used in Artworks

**DOI:** 10.3390/molecules27051475

**Published:** 2022-02-22

**Authors:** Sara Mattana, Alice Dal Fovo, João Luís Lagarto, Maria Chiara Bossuto, Vladislav Shcheslavskiy, Raffaella Fontana, Riccardo Cicchi

**Affiliations:** 1 National Institute of Optics—National Research Council (CNR-INO), Largo E. Fermi 6, 50125 Florence, Italy; sara.mattana@ino.cnr.it (S.M.); alice.dalfovo@ino.cnr.it (A.D.F.); raffaella.fontana@ino.cnr.it (R.F.); riccardo.cicchi@ino.cnr.it (R.C.); 2 Biophotonics Platform, Champalimaud Clinical Centre, Champalimaud Foundation, Av. Brasilia, 1400-038 Lisbon, Portugal; 3 Department of Physics, University of Florence, Via G. Sansone 1, 50019 Sesto Fiorentino, Italy; mariachiara.bossuto@gmail.com; 4 Becker & Hickl GmbH, Nunsdorfer Ring 7-9, 12277 Berlin, Germany; vis@becker-hickl.de; 5 Privolzhskiy Research Medical University, 603005 Nizhny Novgorod, Russia; 6 European Laboratory for Non-Linear Spectroscopy (LENS), Via Nello Carrara 1, 50019 Sesto Fiorentino, Italy

**Keywords:** time-resolved fluorescence imaging, fluorescence lifetime imaging, TCSPC, phasor analysis, Gaussian mixture model, pigments, binders, cultural heritage

## Abstract

The non-invasive analysis of fluorescence from binders and pigments employed in mixtures in artworks is a major challenge in cultural heritage science due to the broad overlapping emission of different fluorescent species causing difficulties in the data interpretation. To improve the specificity of fluorescence measurements, we went beyond steady-state fluorescence measurements by resolving the fluorescence decay dynamics of the emitting species through time-resolved fluorescence imaging (TRFI). In particular, we acquired the fluorescence decay features of different pigments and binders using a portable and compact fibre-based imaging setup. Fluorescence time-resolved data were analysed using the phasor method followed by a Gaussian mixture model (GMM) to automatically identify the populations of fluorescent species within the fluorescence decay maps. Our results demonstrate that this approach allows distinguishing different binders when mixed with the same pigment as well as discriminating different pigments dispersed in a common binder. The results obtained could establish a framework for the analysis of a broader range of pigments and binders to be then extended to several other materials used in art production. The obtained results, together with the compactness and portability of the instrument, pave the way for future in situ applications of the technology on paintings.

## 1. Introduction

A fluorescence emission is one of the two subcategories of photo-induced luminescence (PL) and is defined as the spontaneous emission of radiation from the singlet excited state of a molecule. The decay time as well as the intensity, spectral features, and polarisation of the fluorescence emission depend on the emitting molecular species and on the local microenvironment [1]. For a considerable time, UV-excited fluorescence has been employed for the non-invasive and qualitative study of works of art as a photographic technique because of its capability of detecting the intrinsic luminescence of the materials employed in the cultural heritage (CH) [2,3,4]. In short, the coatings and protective varnishes as well as other restoration materials that are invisible to the naked eye can be disclosed based on the spectral features of their fluorescence emission following illumination with a UV source, e.g., a low-pressure mercury lamp [5]. More recently, spectrally resolved laser-induced fluorescence (LIF) has made it possible to differentiate between various organic and inorganic materials such as oils, waxes, varnishes, resins, proteins, and pigments [6,7,8,9,10]. 

Although PL techniques have long been applied in the CH field, most studies are limited to steady-state measurements. In such cases, the identification of fluorescent materials based only on the emitted spectra can be complex and misleading, making it necessary to apply other analytical techniques [11]. Different molecules can emit signals with similar spectral characteristics, whereas chemical modifications of the bulk materials or competing optical phenomena (self-absorption or scattering) as well as the molecular environment can produce slightly different spectra [8,12,13]. Moreover, the emission of proteins and oil-based organic binders can be strongly affected by the presence of non-fluorescent pigments, often generating false-negative results due to quenching or optical absorption effects [9,12,14]. Therefore, the application of fluorescence spectroscopy in the CH field may be limited, especially for the characterisation of coating and binding materials. As reported in [15,16], the emitted signal of complex organic molecules such as proteins and fatty acids, commonly found in binders and protective compounds, can be more efficiently interpreted by analysing their fluorescence emission dynamics.

Time-resolved fluorescence imaging (TRFI) and spectroscopy aim to bring out the specific characteristics of fluorescence spectra by resolving the decay dynamics of fluorophores characterised by overlapping emission spectra but with distinct fluorescence lifetimes. In particular, TRFI can be useful for the spatial mapping of heterogeneous materials, benefiting from the fact that the fluorescence lifetime is, to an extent, relatively unaffected by changes in the fluorescence intensity and in the concentration of the emitted materials [17]. The characteristic fluorescence lifetimes of several natural and synthetic organic materials such as binders, waxes, and varnishes, as well as of inorganic pigments such as zinc white, titanium white, chrome yellow, cadmium-based pigments, ultramarine, and cinnabar, have been studied with time-resolved fluorescence techniques in waxes, resins, binders, or glues [13,18,19], showing a decay time ranging from a picosecond to microseconds. The decay kinetics of long-chain organic molecules are highly influenced by external factors such as temperature, molecular flexibility, and pH [13,18,19] as well as by the complex chemical nature of the materials used in artworks. A multimodal approach based on the application of other analytical techniques such as Raman, infrared spectroscopies, or X-ray fluorescence [5,20,21,22,23] would be useful to remove any ambiguity as well as for a proper interpretation of the data obtained from fluorescence measurements. As described in [23], a preliminary investigation to discriminate luminescent organic and inorganic materials through the study of their fluorescence lifetimes was performed using a new fibre-based TRFI setup designed for biomedical applications [24,25]. Several binders and coatings, pure or pigmented, spread on glass and metallic supports were analysed, showing a possible correlation of the fluorescence lifetime with the viscosity of the binder [23]. It is well-known that in pigmented mixtures, the fluorescence signal is mainly influenced by the pigments, which can be responsible for several competing phenomena such as the absorption of fluorescence, optical scattering, and chemical interactions [12,13,14]. 

In this study, we investigated the influence of binders on the fluorescence lifetime of pigments and we were able to determine variations in the fluorescence lifetimes of pigments when mixed with different binders. In more detail, time-correlated single photon counting (TCSPC) was used for TRFI measurements because of its higher sensitivity, dynamic range, and temporal resolution in comparison with other time-resolved methods. The phasor approach was applied to process the TRFI data using the Fourier transform of the fluorescence signal, obtaining the polar coordinates *g* and *s* from which the characteristic fluorescence lifetime τ-phase was calculated. For the first time, to the best of our knowledge, the Gaussian mixture model (GMM) was employed to analyse the fluorescence lifetime data obtained from the artistic materials. This method addressed the need for the unsupervised and automatic identification of fluorescence species within a sample. The combined phasor and GMM approach lays the groundwork for a faster and an automated analysis of the many different materials used in artworks.

## 2. Materials and Methods

### 2.1. Samples

**Figure 1:**
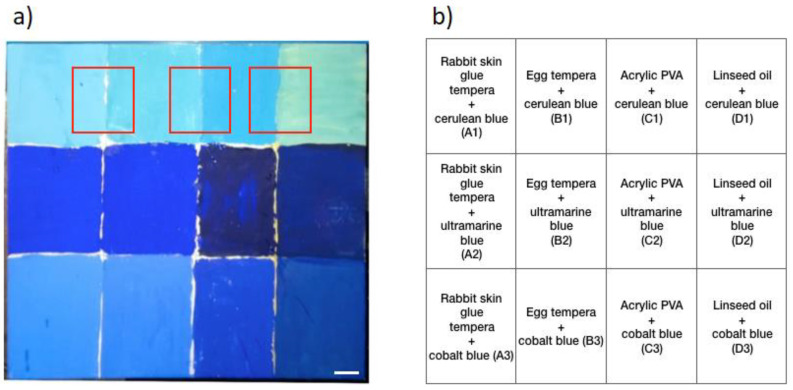
The 15 × 15 cm^2^ wood support divided into 12 areas, 1 for each mixture. (**a**) Image and (**b**) schematics of pigments (one on each row) and binders (one on each column) mixed together. The red squares correspond with the areas of interest in between two different mixtures considered in our analysis. Scale bar (white): 1 cm.

**Figure 2:**
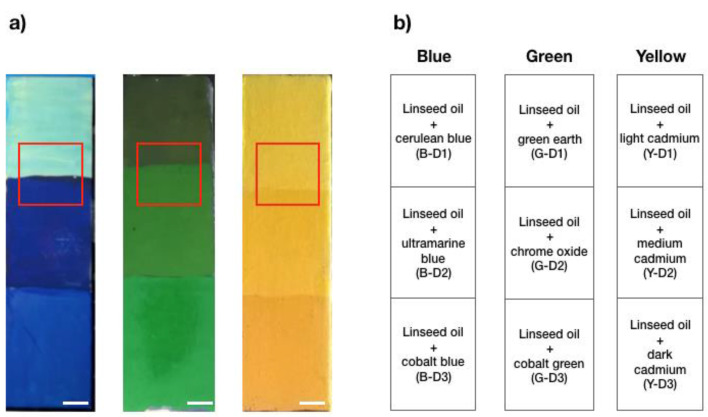
The 3.75 × 15 cm^2^ wood (blue) and canvas (green and yellow) supports divided into 3 areas each on which the mixtures were spread. (**a**) Image and (**b**) schematics of the three pigments mixed with the same binder, i.e., linseed oil. The blue, green, and yellow squares correspond with the areas of interest considered for our analysis. Scale bar (white): 1 cm.

The samples analysed in this work were provided by the Museo de Bellas Artes de Asturias, Spain, and comprised several pigments and binders typically used in artworks. More specifically, nine different pigments comprising three blues (cerulean blue, ultramarine blue, and cobalt blue), three yellows (light cadmium yellow, medium cadmium yellow, and dark cadmium yellow), and three greens (green earth, chrome oxide, and cobalt green) (Kremer Pigmente GmbH & co KG., Aichstetten, Germany) mixed with four different binders—namely, rabbit skin glue, egg yolk, acrylic polyvinyl acetate (PVA), and linseed oil—were spread on wood or textile supports. The blue sample was 15 × 15 cm^2^ divided into 12 areas; the green and yellow samples were 3.75 × 15 cm^2^, divided into 3 areas each. The blue sample was prepared as a chessboard in which every row corresponded with the same pigment mixed with different binders. Each column showed the mixture of the different pigments with the same binder. Each area was tagged with an acronym, as reported in Figure 1 and Figure 2.

Every wood or textile support was prepared for the painting layer with rabbit skin glue and rock alum diluted in water, and with two superimposed layers of rabbit skin glue, rock alum, and calcium carbonate diluted in water. The samples were not varnished.

### 2.2. Setup 

The experimental setup used for the TRFI measurements is described in detail in the literature [23,24,25]. Briefly, it consists of a picosecond pulsed laser diode excitation source at 375 nm (BDL-SMN-375, Becker & Hickl GmbH, Berlin, Germany) operated at 20 MHz. The excitation light, together with a 660 nm aiming beam from a fibred light emitting diode (LED, M660FP1, Thorlabs, Newton, NJ, USA), superimposed on the excitation beam as a visual reference for the measurements, was delivered to the sample by two 200 μm core diameter fibres (0.22 NA). The tip of the fibre bundle was handheld and could be freely moved over the sample surface. The distance between the fibre and the sample was maintained between 2 and 10 mm during the measurements, resulting in an irradiated spot diameter between 0.9 and 4.5 mm. The fluorescence emitted from the sample was collected by the fibre bundle, narrowed by an emission filter (FF01-470/28-25 or FF01-534/20-25, Semrock, Rochester, NY, USA) to the 470 ± 14 nm band (blue sample) or the 534 ± 10 nm band (green and yellow samples) and then collected by a detector (HPM-100-40, Becker & Hickl GmbH) connected to a TCSPC acquisition card (SPC-730, Becker & Hickl GmbH). The TCSPC card was configured to an 8-bit resolution corresponding with 256 bins at 195 ps per bin within a 50 ns window. The average laser power on the sample was kept below 10 μW for all the measurements. To provide bright illumination of the specimen field of view (FOV) during the acquisitions, the setup also included a white LED (LED, MNWHL4, Thorlabs, Newton, NJ, USA), which was driven out-of-phase with the TCSPC signal to permit fluorescence measurements to be obtained under a bright background light. Finally, a USB colour camera of 640 × 480 pixels (DFK33UP1300, The Imaging Source, Bremen, Germany) was used to record the measurements. 

### 2.3. Analytical Procedures

The fluorescence intensity decays collected from the samples were analysed using the phasor method, which is becoming a widely employed approach for interpreting fluorescence lifetime data due to its fit-free nature and relatively straightforward interpretation, which makes it suitable for a real-time implementation. 

A more detailed description of the phasor method is available in the literature [23,26,27,28]. The phasor approach quickly characterises the fluorescence decays and, for this reason, is suitable for a real-time analysis. In this application, every decay acquired from a group of pixels was segmented to generate binary fluorescence lifetime maps. In more detail, the coordinates (*g* and *s*) of each fluorescence decay obtained from the phasor analysis corresponded with a single point (phasor) on the phasor plot. When N fluorescence decays were acquired during an acquisition session, the corresponding N phasors formed a cluster in the phasor domain. Hence, each point in the phasor cluster, plotted in a universal semicircle, could also be traced back to a group of pixels in the fluorescence lifetime image due to the reciprocity between the fluorescence decay and the phasor transformation. For each decay curve, a characteristic phase lifetime, the τ-phase, could be determined following the expression $\tau_{phase}=s/\left(2\pi fg\right)$, where *f* is the repetition rate of the laser at 20 MHz.

In this work, a threshold was applied to all the datasets; only decays with >100 peak counts were considered during the phasor plot analysis in order to avoid artefacts and over and/or underestimations of the τ-phases.

Gaussian mixture models (GMMs) are commonly employed in data mining, machine learning, pattern recognition, and statistical analyses. A comprehensive description of this method is provided elsewhere [29,30]. In brief, a GMM is a clustering technique that assumes all data points can fit to a linear combination of a finite number of Gaussian distributions. This model consists of a weighted sum of component Gaussian densities with the number of terms K being the number of clusters, as reported in Equation (1):

(1) $$p\left(x\right)=\overset{K}{\underset{i=1}{\sum }}f_{i}N\left({µ}_{i},\Sigma_{i}\right)$$

where *f_i_* is the fraction of each cluster, *N*(*µi*, Σ*i*) is the multivariate normal distribution with two parameters characteristic of the cluster data, and *µi* and Σ*i*, are the mean coordinates and covariance matrix, respectively. 

This model was implemented by means of the MATLAB (The Mathworks Inc., Natick, MA, USA) function fitgmdist, which uses the expectation–maximization (EM) algorithm to iteratively optimise the model until a convergence is obtained. This approach is based on altering the expectation and maximisation phases as follows. A few values for the parameters are considered as a starting point. At each iteration, the likelihood belonging to each cluster, i.e., the expectation, is then obtained by evaluating the data points under the current model. Finally, the likelihood is employed to obtain the mean position and the covariance matrix of every cluster, weighting the points toward the subsequent maximisation.

**Figure 3:**
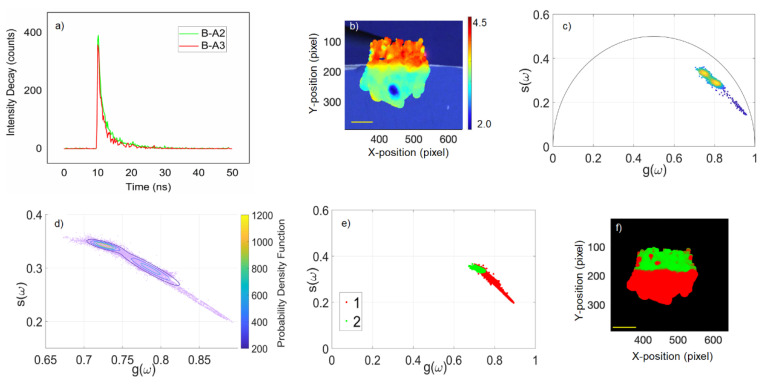
Example of data analysis using a GMM approach. (**a**) Representative fluorescence decays: the green curve is from the ultramarine blue + rabbit skin glue mixture (sample B-A2) and the red curve is from the cobalt blue + rabbit skin glue mixture (sample B-A3); (**b**) white light image of the interface of samples B-A2 and B-A3 augmented with the τ-phase map obtained after the phasor analysis. τ-phase colour bar scale range is from 2.0 ns (blue) to 4.5 ns (red); (**c**) phasor plot obtained from fluorescence lifetime measurements of the interface of sample B-A2/B-A3. In this example, the data produce two clusters of phasors; (**d**) ellipses representing the points that belong to each cluster after a GMM analysis; (**e**) segmentation of the phasor plot cloud into two different clusters (cluster 1 in red and cluster 2 in green); (**f**) segmented cluster map obtained after a GMM analysis corresponding with the same area of interest shown in (**a**). Scale bar (yellow): 5 mm.

An important point of the GMM approach consists of estimating the number of clusters K employed to model the datasets, which is often considered to be a downside. In several cases, when the number of species in the dataset is unknown, the k-means algorithm has been used to estimate the number of clusters [30,31,32,33]. In our case, the fluorescence measurements were carried out in the boundaries of two known mixtures of pigments and binders. We assumed, therefore, that each mixture formed a cluster in the phasor plot, i.e., K = 2 in each dataset. An example of this method is shown in Figure 3 in which the GMM analysis was performed on the interface of two different mixtures of ultramarine blue and cobalt blue in animal glue (B-A2 and B-A3 of Figure 1a). Figure 3a shows two typical fluorescence decays in green that form the ultramarine blue + rabbit skin glue mixture area; in red is the cobalt blue + rabbit skin glue mixture area. Figure 3b shows the white light image of the sample with the fluorescence lifetime map with the coding for the τ-phase superimposed on it. The phasor plot in Figure 3c is a 2D density scatter plot in which the density colour scale corresponded with the number of decays with the same *g* and *s* coordinates. The data showed two regions with a high density of phasors that were closely located. The phasor *g* and *s* coordinates were used as inputs for the GMM algorithm, which modelled the data to assign a probability of each phasor cluster. Considering the list of probabilities, the option adopted was to cut hard edges, meaning that at each coordinate, the cluster with the higher probability was chosen and then each point was assigned to a cluster. Figure 3d shows the ellipses of the points that belonged to the two clusters using this approach plotted in a probability density colour-coded scale. In Figure 3e, a colour is assigned to each cluster (red for cluster 1 and green for cluster 2). As each point in the phasor cloud could be traced back to a position in the fluorescence map, we could identify the spatial location of each cluster in the white light image, as demonstrated in the segmented image in Figure 3f.

## 3. Results

In order to better interpret the fluorescence signals emitted by pigments and binders, TRFI measurements were acquired on different samples divided into different areas. A mixture composed of a pigment and a binder was spread on each area. 

**Figure 4:**
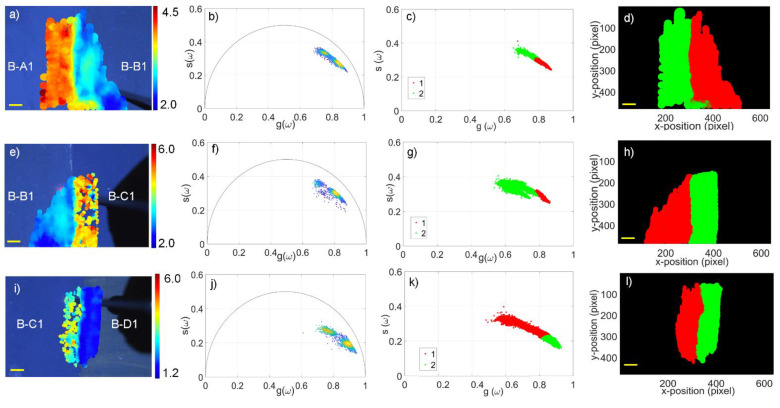
Results of time-resolved fluorescence imaging, phasor, and GMM analysis of cerulean blue pigment mixed with the four binders. The first column of panels from the left shows the white light images of the areas of interest and the τ-phase maps superimposed on (**a**) cerulean blue + rabbit glue (left) and egg yolk (right); (**e**) cerulean blue + egg yolk (left) and acrylic PVA (right); and (**i**) cerulean blue + acrylic PVA (left) and linseed oil (right). The panels of the second column (**b**,**f**,**j**) represent the related phasor plots, showing two clusters each; the plots on the third column (**c**,**g**,**k**) show the clustering of the phasor clouds after the GMM analysis. The last column on the right (**d**,**h**,**l**) shows the segmented cluster maps obtained after the GMM analysis corresponding with the same areas of interest of the samples in (**a**,**e**,**i**). Scale bar (yellow): 5 mm.

**Figure 5:**
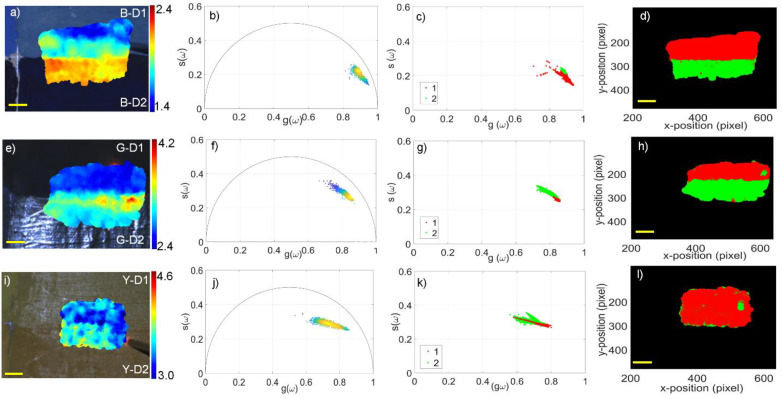
Results of time-resolved fluorescence imaging, phasor, and GMM analysis for linseed oil used as a binder and mixed with two different pigments in each sample. The first column of panels from the left shows the white light images of the areas of interest and the τ-phase maps superimposed on (**a**) linseed oil + cerulean blue (upper) and ultramarine blue (lower); (**e**) linseed oil + green earth (upper) and chrome oxide (lower); and (**i**) linseed oil + light cadmium yellow (upper) and medium cadmium yellow (lower). The panels of the second column (**b**,**f**,**j**) represent the related phasor plots, showing larger and more dispersed single clusters; the plots on the third column (**c**,**g**,**k**) show the clustering of the phasor cloud after the GMM analysis. The last column on the right (**d**,**h**,**l**) shows the segmented cluster maps obtained after the GMM analysis corresponding with the same areas of interest of the samples in (**a**,**e**,**i**). Scale bar (yellow): 5 mm.

The first sample was composed of three different pigments (one per row) mixed with four binders (one per column), as schematised in Figure 1b. The TRFI acquisitions were performed over the region at the interface between two mixtures in order to highlight the differences in the fluorescence lifetime of the mixtures made of the same pigment dispersed in different binders (Figure 1) and the mixtures of different pigments dispersed in the same binder (Figure 2). We obtained the τ-phases of each mixture, as reported in Table 1. However, we showed only one example of the phasor analysis and the GMM approach for each specimen (Figure 4 and Figure 5).

The data were first processed through the phasor method, obtaining a τ-phase as an average of 3000 acquisition points for each measurement with an integration time of 15 ms each for a total acquisition time of 45 s for each map. For each sample, the phasors were typically found to aggregate in two regions within the phasor plot (see Figure 4, panels b, f and j), which could be intuitively attributed to each mixture of pigments and binders in the sample. To identify the cluster to which each phasor point pertained in an automatic and unsupervised manner, we employed the GMM method (using K = 2 clusters) directly to the *g* and *s* phasor coordinates. Red and green colours were used to distinguish the two clusters in the phasor plot (Figure 4, panels c, g and k) and the same colours represented the two populations on the spatial images of the samples (Figure 4, panels d, h and l). This method could also help to classify different populations in the case of very close phasor clouds or a single large distribution (Figure 5). Finally, the mean τ-phases and their dispersions were obtained by calculating the mean τ-phase of each area, namely, of each population and its standard deviation (see Table 1).

### 3.1. Discrimination of Binders

As an example of the fluorescence lifetime analysis of mixtures with the same pigment and different binders, the first row of Figure 1a was considered. Cerulean blue pigment was mixed with the four binders, spreading each mixture close to the other (see Figure 1b). 

Figure 4a,e,i shows the white light images augmented with the TRFI maps of the τ-phases of the B-A1/B1, B-B1/C1, and B-C1/D1 areas, pointing out the different lifetimes of the diverse binders. Although the acrylic binder showed greater inhomogeneity, rabbit glue and acrylic PVA had the same τ-phase (3.7 ns). Linseed oil had the shortest and most uniform τ-phase (1.7 ns), whereas the egg yolk showed a τ-phase value in between the others (2.8 ns).

Figure 4b,f,j shows the phasor plots related to the fluorescence lifetimes maps, revealing two distinct phasor clouds. Figure 4c,g,k shows the results of the GMM analysis, confirming the presence of two different clouds for each phasor plot that belonged to two diverse populations, one colour-coded in red and the other in green. The same colour-coded division is shown in Figure 4d,h,l. The two populations were displayed in the spatial images, illustrating where the two clusters of the phasor plot were spatially distributed. This evidence was further confirmed by examining the other blue pigment results, as reported in Table 1.

### 3.2. Discrimination of Pigments

Figure 5 shows the results obtained from the linseed oil paints made with six blue, green and yellow pigments, i.e., cerulean blue, ultramarine blue, green earth, chrome oxide, and light and medium cadmium yellow, which were selected due to the homogeneity of the layers (Figure 2).

In this case, white light images augmented with fluorescence lifetime maps and corresponding phasor plots were also obtained. However, looking at the phasor dispersions in Figure 5b,f,j, rather than two distinct clouds as in the previous case, a larger and a more dispersed single cloud was evidenced.

Concerning the TRFI maps, we noticed that the τ-phases obtained in the blue and green samples, namely, B-D1/B-D2 and G-D1/D2 (Figure 5a,e), were slightly different, showing values of 1.7–2.1 ns and 2.5–2.9 ns, respectively; in the third map (Figure 5i), the yellow paints, namely, Y-D1/D2, showed more similar τ-phases (3.8 and 3.6 ns, respectively). The GMM analysis, displayed in Figure 5c,g, indicated the presence of two clusters corresponding with the two different pigmented areas as reported in Figure 5d,h. However, in the case of the yellow pigments, the lifetime differences were too small to be detected employing the GMM approach (Figure 5k,l).

## 4. Discussion

In addition to the widely documented influence of pigments on the fluorescence signal of the binder in mixtures [12,13,14], our results showed how different binders affected the fluorescence lifetimes of pigments. In particular, we found that mixtures containing rabbit glue and PVA as binders had apparently similar values and, more generally, longer lifetimes compared with the other paints (Table 1 and Figure 4). However, PVA-based mixtures showed higher standard deviations due to generally weaker fluorescence signals that resulted in a poor signal-to-noise ratio (SNR). Mixtures of linseed oil and blue pigments were characterised by very intense fluorescence decays and more homogeneous maps with τ-phases affected by very small standard deviations. Starting from the slight differences in the τ-phases of the latter mixtures, we performed an analysis of the data of the green + linseed oil mixtures and observed a high fluorescence intensity but subtle differences in the τ-phases, probably due to a higher fluorescence contribution from the binder. This result was also evident in the yellow samples (see Figure 5), even if the fluorescence decays of the three cadmium pigments showed a lower intensity than the blues or greens. This was probably due to the laser excitation wavelength (375 nm), which excites more efficiently the blue and green pigments than yellow and red pigments. For a broader application of the setup and methods described here, multiple excitation sources could be employed in order to cover the broad spectral range of the fluorescence emission of the pigments used in works of art. Although the GMM failed in clustering the decay data in the case of the yellow pigments, the consistency of the measured τ-phase values demonstrated that the decay values were strongly affected by the binder.

In this work, we investigated the suitability of fluorescence lifetime data processed with the phasor and GMM methods to discriminate pigments and binders in painted mixtures. On one hand, the phasor analysis on the TRFI maps proved to be a powerful tool for differentiating the binders and pigments in real-time, avoiding a longer and operator-dependent fitting analysis. On the other hand, the GMM approach automated the clustering of the phasor transformed data. The advantage of this technique was the possibility of automatically segmenting the images in the phasor domain rather than manually selecting each region in the real space domain. This automatic approach significantly improved the reproducibility of the experiment and the reliable extraction of quantitative and impartial data from the experimental dataset. In this way, several imaging datasets could be automatically analysed, making the GMM a valuable tool for a TRFI data analysis in the phasor domain. The use of chromatically contrasting colours for the clusters was the simplest way to highlight the GMM results; however, this approach applied a probability of each pixel of the image to each coordinate of the phasor plot highlighting a quantitative feature. This allows the realisation not only of binary images such as those presented in this paper but also two- or multiple-colour images with colour tones representing the combination of probability calculated by the GMM. Such results pave the way for future applications of this method to other substances commonly used in works of art such as varnishes and resins. 

## 5. Conclusions

This study demonstrated that the TRFI technique could discriminate the pigments and binders in several mixtures based on the τ-phase maps. The portability and compactness of our fibre-based TRFI setup, together with the real-time data processing offered by the two analytical approaches, make fibre-based TRFI a powerful technique for measuring various materials used in works of art. In particular, a real-time phasor plot analysis avoids the use of time-consuming fitting routines and the GMM approach opens the route to an automated operator-independent segmentation of several fluorescence decay datasets. The experimental setup could be further complemented by other analytical techniques such as Raman spectroscopy [34] in order to discriminate different species despite uncertainties due to very slight differences between the measured τ-phases. The obtained results established a framework for the future application of the technique in the field of cultural heritage by analysing a broader range of pigments and binders as well as varnishes and resins. Further, this work paves the way for a future in situ application of fibre-based TRFI investigations on paintings not only for the material identification but also for discriminating between original and retouched areas based on the different τ-phases measured over the painted surface.

## Figures and Tables

**Table 1:** List of pigments and binders of the blue, yellow, and green samples, their emission wavelengths, and the mean τ-phases of their mixtures (for n = 3000 acquisitions).

Pigment	Binder	Acronym	Emission Wavelength	Mean τ-Phase ± SD (ns)
Cerulean Blue	Rabbit glue	B-A1	456–484 nm	3.7 ± 0.3
	Egg yolk	B-B1		2.8 ± 0.5
	Acrylic PVA	B-C1		3.7 ± 0.8
	Linseed oil	B-D1		1.7 ± 0.3
Ultramarine Blue	Rabbit glue	B-A2		3.9 ± 0.3
	Egg yolk	B-B2		3.4 ± 0.4
	Acrylic PVA	B-C2		4.5 ± 0.8
	Linseed oil	B-D2		2.1 ± 0.2
Cobalt Blue	Rabbit glue	B-A3		3.0 ± 0.2
	Egg yolk	B-B3		2.8 ± 0.2
	Acrylic PVA	B-C3		3.1 ± 1.3
	Linseed oil	B-D3		1.9 ± 0.1
Green Earth	Linseed oil	G-D1	524–544 nm	2.5 ± 0.1
Chrome Oxide		G-D2		2.9 ± 0.3
Cobalt Green		G-D3		2.9 ± 0.2
Light Cadmium Yellow		Y-D1		2.8 ± 0.2
Medium Cadmium Yellow		Y-D2		3.8 ± 0.5
Dark Cadmium Yellow		Y-D3		3.6 ± 0.8

## Data Availability

The acquired data are available for research purposes upon request to the authors.
